# 

**Published:** 1896-02-22

**Authors:** 


### NOTICE TO CORRESPONDENTS.

All MS., Letters, Books for Review, and other matters intended for the Editor should be addressed THE EDITOR, THE LODGE, PORCHESTER SQUARE, LONDON, W.

The Editor cannot undertake to return rejected MS., even when accompanied by stamped directed envelope.

### The Student of Medicine.

### APPOINTMENTS.

J. S. Aitken, L.R.C.P.Edin., L.F.P.S.Glas., appointed Medical Officer for the Shap District and the Workhouse of the West Ward Union. A. E. Barker, F.R.C.S., appointed Examiner in Surgery at the Victoria University. R. A. S. Eden, M.B., C.M. Aberd., D.P.H., appointed Medical Officer for the Norton Canes District of the Cannock Union. R. C. Elsworth, M.B., C.M.Edin., M.R.C.S.Eng., appointed Visiting Surgeon to the Swansea Hospital. W. E. Fothergill, M.A.Edin., B.Sc., M.B., appointed Medical Officer for the out-district of Moss Side of the Chorlton-upon-Medlock Dispensary. T. W. Garstang, M.A. Oxon., M.R.C.S.Eng., appointed Medical Officer of Health to the Northwich Urban District Council. J. R. Green, D.Sc., F.R.S., appointed Examiner in Botany at the Victoria University. Mr. E. Haines, appointed Junior Medical Officer to the Wandsworth and Clapham Union Infirmary. F. Harvey, M.R.C.S. Eng., L.S.A., appointed Medical Officer of Health to the Padstow Urban District Council. Mr. E. H. Haskett, appointed Medical Officer for the Southowram District of the Halifax Union. Dr. G. Jackson, reappointed Medical Officer of Health to the Compton Gifford Urban District Council. W. M. Jennings, B.A.Dubl., M.B., B.Ch., appointed Medical Officer of Health to the Dore Rural District. R. Jones, M.D., C.M.Edin., D.P.H.Camb., appointed Medical Officer of Health for the Urban District of Festiniog. H. W. Kendall, M.R.C.S., L.R.C.P.Lond., appointed Assistant Surgeon to the Bristol Hospital for Sick Children and Women. W. R. Martine, M.B.Edin., C.M., appointed Physician to the Western District Combination Fever Hospital, Haddington, and District Medical Officer of Health for the Parishes of Athelstaneford, Morham, and Yester. C. D. Morris, L.R.C.P. Lond., M.R.C.S.Eng., reappointed Medical Officer of Health to the Sunbury District Council. R. Nitch-Smith, M.R.C.S., L.R.C.P.Lond., appointed Senior House Physician to the Westminster Hospital. G. H. Oliver, L.R.C.P.Lond., M.R.C.S.Eng., appointed Medical Officer of Health to the Clayton Urban District. E. G. Peck, M.A.Cantab., L.R.C.P.Edin., M.R.C.S.Eng., appointed Medical Officer for the Queensbury District of the Halifax Union. C. Perrott, L.R.C.P., L.R.C.S.I., appointed Medical Officer of Health to the Kingswood Urban District. A. R. Rackham, L.R.C.P.Edin., L.M., M.R.C.S.Eng., appointed Medical Officer to the Mitford and Launditch Union Workhouse. J. H. Renshaw, M.B., Ch.B.Vict., appointed House Surgeon to the Manchester Royal Infirmary. J. A. Rigge, L.R C.P.Lond., M.R.C.S.Eng., appointed Medical Officer for the Henley District of the Henley Union. E. A. Schafer, F.R.S., M.R.C.S., reappointed Examiner in Physiology at the Victoria University. W. A. Sharpin, L.R.C.P.Lond., M.R.C.S., appointed Resident Medical Officer to the Royal South Hants Hospital, Winchester. Mr. F. B. G. Stapleford, appointed Assistant Medical Officer to the Aston Union Workhouse. Dr. Stephenson, appointed Medical Officer for the No. 1 District of the Carrick-on-Suir Union. N. I. C. Tirard, M.D., F.R.C.P., appointed Examiner in Materia Medica and Pharmacy and in Pharmacology and Therapeutics at the Victoria University. C. A. Wickham, L.R.C.P., L.R.C.S. Edin., L.F.P.S.Glasg., appointed Medical Officer for the Fourth District of the East Ashford Union. M. A. Wood, L.R.C.P. Lond., F.R.C.S.Eng., appointed Medical Officer of Health to the Leadbury Urban Sanitary District.

### VACANCIES.

### Resident Medical Officers.

The following vacancies are announced this week, the amount of salary in each case being given after the name of the institution :—

Belgrave Hospital for Children, 79, Gloucester Street, S.W.—House Surgeon. Board, lodging, light, and fuel found. Applications to the Secretary by February 29th.

Buckingham General Infirmary, Aylesbury.—Surgeon and Apothecary; doubly qualified. Salary £80 for the first year, increasing £10 annually to £100, with board and lodging, washing, coals, and candles in furnished apartments. Applications to Mr. George Fell, Solicitor, Aylesbury, by February 24th.

Cumberland Infirmary, Carlisle.—Assistant House Surgeon. Appointment for one year. Salary £40 per annum, with board, lodging, and washing. Applications to the Secretary by February 25th.

East London Hospital for Children, Glamis Road, Shadwell, E.—House Physician; must be duly qualified. Board, Lodging, &c., provided, but no salary. Applications to Thomas Hayes, Secretary, by February 29th.

Lincoln County Hospital.—Assistant House Surgeon. Appointment for six months, but eligible for re-election. Honorarium £10 for each period of six months, with board and washing. Applications to the Secretary by February 22nd.

Royal Free Hospital, Gray's Inn Road, W.C.—Resident Medical Officer (House Physician); doubly qualified. Appointment for six months, but eligible for re-election. No salary, but board, residence, and washing provided by the hospital. Applications to the Secretary by February 29th.

Salford Royal Hospital.—House Surgeon. Salary £100 per annum, with board and residence. The Junior House Surgeon is a candidate, and, in the event of his being appointed, there will be a vacancy in the post of Junior House Surgeon. Salary £50 per annum, with board and residence. Applications to the Secretary by February 22nd.

Sheffield Union.—Resident Assistant Medical Officer for the Workhouse, Fir Vale, Pitsmoor. Salary £100 per annum, with apartments, rations, and other usual allowances. Single or widower. Applications to Joseph Spencer, Clerk to the Guardians, Union Offices, Sheffield, by February 26th.

University College, London.—Professorship of Pathology. Applications to the Secretary, J. M. Horsburg, M.A., by February 29th.

### Non-Resident Medical Officers.

Dental Hospital of London, Leicester Square.—Assistant Dental Surgeon. Applications to J. Francis Pink, Secretary, by March 9th.

Kent and Canterbury Hospital, Canterbury.—Dental Surgeon. Applications to the Secretary by February 28th.

London Hospital, Whitechapel, E.—Two Surgeon Dentists. Applications to the House Governor by February 28th.

Just Published. Third Edition. Re-written and much enlarged. Illustrated with nearly 50 Plans (mostly new ones), Diagrams, &c. Crown 8vo. Cloth gilt. Price 10s. 6d.

### COTTAGE HOSPITALS:

GENERAL, FEVER, AND CONVALESCENT.

**Their Progress, Management, and Work in Great Britain and Ireland, and the United States of America.**

With an Alphabetical List of Every Cottage Hospital at Present Opened.

BY HENRY C. BURDETT,

Author of "Hospitals and Asylums of the World," "Burdett's Hospitals and Charities," &c., &c.

This new Edition is issued in response to the great demand which has existed for the book during the past few years, and the wide interest it has excited amongst many classes of readers, both in this country and the United States. The Second Edition was published more than 15 years ago.

"An invaluable book of information on a subject which attracts increasing attention. . . . . A complete manual of all that relates to the founding, the cost, and the management of cottage hospitals. It embodies the results of wide experience, and it demonstrates the methods by which efficiency and economy may be secured. . . . . . No more complete manual could be required. It is a pleasure to see the care that has been bestowed on the work, and to bear testimony to its utility; few half-guineas could be better spent than in its purchase."—*St. James's Gazette.*

"Contains all the information which could be required by anyone who undertakes to found or to manage a cottage hospital."—*British Medical Journal.*

Demy 8vo., with Two Plates, cloth gilt, over 400 pp. Price 12s. 6d. net.

### MEDICAL HISTORY FROM THE EARLIEST TIMES,

By E. T. WITHINGTON, M.A., M.B. Oxon.

"One of the best attempts that has yet been made in the English language to present the reader with a concise epitome of the history of medicine, and as such we very cordially commend it."—*Glasgow Medical Journal.*

"We cordially recommend the History of Medicine to the attention of every true lover of its science and its art."—*Dublin Medical Journal.*

"A not unwelcome contribution to a neglected department of medical literature."—*Provincial Medical Journal.*

London: THE SCIENTIFIC PRESS (Limited), 428, Strand, W.C.

### BLACKIE AND SON'S

### ELEMENTARY TEXT-BOOKS.

*FULLY ILLUSTRATED.*

**ELEMENTARY PHYSIOLOGY.** By Professor AINSWORTH DAVIS. With Appendix for Agricultural Students. Cloth, 2s.

"Written in a clear, forcible style, and crammed with detailed information which is arranged with great method. The illustrations are profuse and admirable, and the directions given to students on practical work are excellent."—*British Med. Journal.*

**AN ELEMENTARY TEXT-BOOK OF PHYSIOLOGY.** By J. M'GREGOR ROBERTSON, M.A., M.B., Lecturer in Physiology Queen Margaret College. New and Revised Edition. Cloth, 4s.

"A good system of arrangement and clear expressive exposition distinguish this book. Definitions of terms are remarkably lucid and exact."—*Saturday Review.*

**ELEMENTARY PHYSIOLOGY.** By VINCENT T. MURCHE. With Coloured Illustrations. Cloth, 2s.

"Its teaching is sound, concise, and up to date, and the care with which the little book is illustrated leaves little to be desired either by teacher or pupil."—*Hospital Gazette.*

**AN ELEMENTARY TEXT-BOOK OF ANATOMY.** By HENRY EDWARD CLARK, Fellow of the Faculty of Physicians and Surgeons of Glasgow. Cloth, 5s.

"The author's experience as a teacher and his reputation as an anatomist are a sufficient guarantee for the general accuracy of its contents. It is methodically arranged, clearly written, and well illustrated."—*Edinburgh Medical Journal.*

**ELEMENTARY HYGIENE.** By H. ROWLAND WAKEFIELD. Cloth, 2s.

"Contains a large amount of information, conveyed in clear and precise terms."—*British Medical Journal.*

London: BLACKIE and SON (Limited), Old Bailey.

Just Out. With numerous Illustrations, demy 8vo., blue buckram, gilt, 3s. 6d.

### DIET IN SICKNESS AND IN HEALTH.

**By Mrs. ERNEST HART,**

*Formerly Student of the Faculty of Medicine of Paris, and of the London School of Medicine for Women.*

With an Introduction by Sir HENRY THOMPSON, F.R.C.S., M.B. Lond.

Sir Henry Thompson, in his Introduction, says: "I do not hesitate to express my opinion that the present volume forms a handbook to the subject, which will not only interest the dietetic student, but offer him, within its modest compass, a more complete epitome thereof than any work which has yet come under my notice."

"The importance of the subject to which Mrs. Ernest Hart has devoted this volume will not be disputed by any practical medical man. As to how Mrs. Ernest Hart has discharged her self-imposed task we prefer to quote from the introduction the words of Sir Henry Thompson, than whom there could be no more competent critic of a subject which he has himself so much illuminated by his admirable essays."—*British Medical Journal.*

*Just Published, 8th Edition. Greatly enlarged and thoroughly revised. Crown 8vo., cloth gilt, 3s. 6d.*

### NURSING: ITS THEORY AND PRACTICE,

BEING A

### COMPLETE TEXT-BOOK OF MEDICAL, SURGICAL, AND MONTHLY NURSING.

**By PERCY G. LEWIS, M.D., M.R.C.S., L.S.A., &c., &c.**

To this edition have been added three chapters on Monthly Nursing, together with additions to the chapters on Blood Diseases, on Antiseptics, on the Nursing of Children, and on Private Nursing. Under these headings will be found a short account of the new Aseptic Treatment, the Antitoxin and Serum Treatments, and the Treatment by Thyroid Extracts.

These additions, combined with the revision throughout of the text, render this work the best and most complete Text-book on Nursing in the market, and strengthen its position as a Classic in the Training Schools of the World.

"We have read the book with interest and can warmly recommend it as a nursing manual. . . . Full of useful hints and information."—*Birmingham Medical Review.*

LONDON: THE SCIENTIFIC PRESS (LIMITED), 428, STRAND, W.C.

### STANDARD TEXT-BOOKS.

**INFANT FEEDING BY ARTIFICIAL MEANS:** A Scientific and Practical Treatise on the Dietetics of Infancy. By S. H. SADLER, Author of "Suggestions to Mothers," "Management of Children," "Education." Ready next week, Second Edition, with a new chapter, and several coloured plates, illustrative of feeding-bottles used in ancient times, &c. Crown 8vo., cloth gilt, with 10 plates and many Illustrations in the text, price 5s. Facsimile Autograph Letters from the late Sir Andrew Clark, M. Pasteur, &c., &c.

"Mrs. Sadler's book deals with the question of the artificial feeding of infants, and contains a very useful collection of the views of the best-known English authorities upon the subject. The truly terrible ignorance displayed by mothers, especially amongst the poor, upon the subject of infant feeding is answerable for an infant mortality so great as to be appalling."—*Daily Chronicle.*

**A PRACTICAL HANDBOOK OF MIDWIFERY.** By FRANCIS W. N. HAULTAIN, M.D. A practical manual produced in a portable and convenient form for reference, and especially recommended for its compactness, conciseness, and clearness. 18mo., profusely illustrated with original cuts, tables, &c.; about 260 pp., handsomely bound in leather, price 6s.

"One of the best of its kind, and well fitted to perform the functions its author claims for it."—*Edinburgh Medical Journal.*

**THE ART OF MASSAGE.** By A. CREIGHTON HALE. A complete Text-book of this valuable art. The most practical, simple, and generally useful work published on the subject. With fully illustrated chapters on the Anatomy of the Human Body. Demy 8vo, nearly 200 pp., cloth gilt, profusely illustrated with over 70 special cuts. Price 6s.

"The latest and most complete book we know on Massage."—*Queen.*

**OPHTHALMIC NURSING.** By SYDNEY STEPHENSON, M.B., F.R.C.S.E., Ophthalmic Surgeon to the North-Eastern Hospital for Children, Shoreditch, E., &c., &c. This is a valuable contribution to Nursing literature on a subject hitherto never handled with special reference to Nursing. On account of its compact and clear arrangement, and its numerous illustrations, it is specially recommended to the use of the Nursing Profession. Crown 8vo, cloth, 200 pp. Profusely illustrated with Original Drawings, with list of Instruments required, and a Glossary of Terms. Price 3s. 6d.

"May very advantageously be studied by nurses and clinical clerks, or dressers, who are about to enter the ophthalmic service of a hospital."—*The Lancet.*

**SURGICAL WARD-WORK AND NURSING.** By ALEXANDER MILES, M.D. (Edin.), C.M., F.R.C.S.E. A practical Manual of Clinical Instruction for Nurses and Students. This book will be found especially useful to beginners, to whom its simple description of elementary principles and treatment in surgery will render it invaluable. Demy 8vo, 200 pp., cloth boards, copiously illustrated. Price 3s. 6d.

"Will furnish much-needed guidance to the student and the nurse in the early stages of their apprenticeship."—*Times.*

**OUTLINES OF INSANITY.** A Popular Treatise on the Salient Features of Insanity. By FRANCIS H. WALMSLEY, M.D., Medical Superintendent of the Darenth Asylum, Metropolitan Asylums Board, Member of Council of Medico-Psychological Association. Demy 8vo, cloth extra, 3s. 6d., or popular edition, stitched, 1s. 6d.

A clearly-written manual in which the various features of mental disorder are fully expounded. The author's style is interesting, and the reader's attention is riveted throughout.

"A most useful little book . . . Many of the descriptive chapters in the body of the work are deeply interesting."—*The Medical Magazine.*

London: THE SCIENTIFIC PRESS (Limited), 428, Strand, W.C.

### "THE HOSPITAL"

AN INDEPENDENT JOURNAL OF

**The Medical Sciences**

AND

**Hospital Administration,**

WITH WHICH IS ISSUED WEEKLY

A SPECIAL

### SUPPLEMENT FOR NURSES.

PUBLISHED EVERY SATURDAY.

PRICE TWOPENCE.

Matrons, Sisters, or Nurses will find in THE HOSPITAL a chronicle of the latest Nursing News, Practical Articles of the highest value to those who wish to keep themselves abreast with the times, and brightly written articles from Nurses all over the world.

SUBSCRIPTIONS CAN COMMENCE AT ANY TIME.

### TERMS OF SUBSCRIPTION.

### WEEKLY ISSUE.

| | Great Britain. | Foreign and Colonial. |
|---|---|---|
| For One Year ... | 10s. 6d. | 15s. 2d. |
| For Six Months | 5s. 3d. | 7s. 7d. |
| For Three Months... | 2s. 8d. | 3s. 10d. |

### MONTHLY PARTS.

| | Post Free to any Part of the World. |
|---|---|
| For One Year | 8s. 6d. |
| For Six Months | 4s. 3d. |
| For Three Months | 2s. 2d. |

Postal Orders and Cheques should be made payable to "The Scientific Press, Limited."

N.B.—In order to prevent delay, all business communications should be addressed to "THE MANAGER" only, and not to any person individually.

### CHANGE OF ADDRESS.

In event of change of address, notice should be forwarded to the Publishers by the Saturday previous to date of publication, and the old address should also be given.

"THE HOSPITAL" can be obtained of all Booksellers and Newsagents in the United Kingdom, and at all the Railway Stalls, of Messrs. W. H. Smith and Son; and also from the following Agents in the Colonies and Abroad:—

PARIS: Librairie Galignani, 224, Rue de Rivoli.
BERLIN: Messrs. A. Asher and Co.
LEIPSIG: Mr. F. A. Brockhaus.
NEW YORK: The International News Co.
MONTREAL: The Montreal News Co.
TORONTO: The Toronto News Co.
MELBOURNE, SYDNEY, ADELAIDE, and ZEALAND: Messrs. Gordon and Gotch.
INDIA: At all the Railway Bookstalls and Agencies of Messrs. A. H. Wheeler and Co.
SOUTH AFRICA: Messrs. Juta, Cape Town.

PUBLISHING OFFICES: 428, STRAND, LONDON, W.C.

### THE 2/6 SERIES OF NURSING MANUALS.

**HOW TO BECOME A NURSE: AND HOW TO SUCCEED.** A complete guide to the nursing profession for those who wish to become Nurses, and a useful book of Reference for Nurses who have completed their training and seek employment. Compiled by HONNOR MORTEN, Author of "The Nurses' Dictionary," &c. Third and Revised Edition. Demy 8vo, 209 pp., profusely Illustrated with Copyright Portraits and Drawings.

"To those who are frequently appealed to by girls, or young women, as to the steps they should take to become nurses, this book of Miss Morten's must prove a perfect godsend."—*British Medical Journal.*

**THE NURSES' DICTIONARY OF MEDICAL TERMS AND NURSING TREATMENT.** Compiled for the use of Nurses, by HONNOR MORTEN. Containing descriptions of the principal Medical and Nursing Terms and Abbreviations, Instruments, Drugs, Diseases, Accidents, Treatments, Physiological Names, Operations, Foods, Appliances, &c., &c., encountered in the Ward or Sick-room. Third and Revised Edition (25th thousand). Demy 16mo, suitable for the apron pocket, handsomely bound in handsome leather, gilt, price 2s. 6d. net, or in cloth, price 2s.

"Should be at the disposal of every nurse."—*Birmingham Medical Review.*

**HOSPITAL SISTERS AND THEIR DUTIES.** By EVA C. LUCKES, Matron to the London Hospital. The favourable reception accorded to the first two editions of this useful and interesting work, and the regular demand for copies of it, encourage the publishers to place this third edition, greatly improved and enlarged, before the Institutions. The experience and position of its author entitle it to authority, and every Matron, Sister, or Nurse who aspires to become a Sister, should read the advice and information contained in its pages. Third Edition. Enlarged and partly rewritten, crown 8vo, cloth gilt, about 200 pp.

**MENTAL NURSING.** A Text-book specially designed for the instruction of Attendants on the Insane. By WILLIAM HARDING, M.D., M.R.C.P. The want of a complete book for the instruction of Asylum Attendants has been long felt, and it is with confidence that the publishers recommend this work to the managers of all Institutions for the Insane. Second Edition. Enlarged, demy 8vo, profusely illustrated, nearly 200 pp., cloth.

**SPINAL CURVATURE AND AWKWARD DEPORTMENT:** Their Causes and Prevention in Children. By Dr. GEORGE MULLER, Professor of Medicine and Orthopædics, Berlin. English Edition, edited and adapted by RICHARD GREENE, F.R.C.P. Crown 8vo, cloth gilt, with many Plates and Illustrations.

"Dr. Müller's little book is an able essay upon the effect produced by exercise, attitude, and movement upon the growth and development of the body. He further gives directions which any intelligent parent or teacher could carry out with the help of very simple apparatus, showing how to avoid or, in early cases, to cure certain malformations which, in the majority of cases, arise either from the neglect of very simple hygienic rules or from carelessness."—*Daily Chronicle.*

**THE MOTHER'S HELP AND GUIDE TO THE DOMESTIC MANAGEMENT OF CHILDREN.** By P. MURRAY BRAIDWOOD, M.D., formerly Senior Medical Officer to the Wirral Hospital for Sick Children. Crown 8vo, cloth gilt.

"The book is altogether admirable."—*Manchester Courier.*

London: THE SCIENTIFIC PRESS (Limited) 428, STRAND, W.C.

*In the Press. Price 5s.*

### BURDETT'S OFFICIAL NURSING DIRECTORY

A DIRECTORY, NOT A REGISTER.

THE HOSPITAL states: "It is well to bear in mind the difference and distinction which exists between a register and a directory. A register implies rights, and suggests that those who are not upon it are in some way or another inferior beings. But a directory is another matter. A directory gives as rights; insertion in it implies no adherence to any particular party or any particular opinion, and in no way interferes with entry on the register of any association, or the list of any institute, to which the individual may belong. All that a directory professes to do is to give information. The 'Medical Register' is not a mere list of qualified medical men; it is admission to the Register, which itself makes him qualified. For years, perhaps even now, 'Churchill's Medical Directory' contained the names of medical men who, so far as public appointments were concerned, were unqualified; it also contains the names of homœopathists, whom four out of every five medical men will not meet; and it certainly contains the names of many for whose moral character we should not like to vouch. But it gives full and accurate information about them all, and for the sake of that information it is constantly consulted both by the public and the doctors."

ACCEPTABLE TO ALL NURSES.

"This ought to be enough to make it acceptable to the public, and every nurse ought to make sure that, whatever list or register she may be on, her name shall also appear on Burdett's 'Official Directory.'" Applications for forms should be made at once to the Editor, "Burdett's Official Nursing Directory," 428, Strand, W.C.

